# A novel three dimensional-printed biomechanically evaluated patient-specific sacral implant in spinopelvic reconstruction after total *en bloc* sacrectomy

**DOI:** 10.3389/fbioe.2023.1153801

**Published:** 2023-05-05

**Authors:** Zhaorui Lv, Jianmin Li, Zhiping Yang, Xin Li, Qiang Yang, Zhenfeng Li

**Affiliations:** ^1^ Qilu Hospital, Shandong University, Jinan, China; ^2^ Cheeloo College of Medicine, Shandong University, Jinan, Shandong, China

**Keywords:** implant, sacral tumor, spinopelvic reconstruction, total *en bloc* sacrectomy, 3D-printed

## Abstract

**Background:** Reconstruction after a total sacrectomy is a challenge due to the special anatomical and biomechanical factors. Conventional techniques of spinal-pelvic reconstruction do not reconstruct satisfactorily. We describe a novel three-dimensional-printed patient-specific sacral implant in spinopelvic reconstruction after total *en bloc* sacrectomy.

**Methods:** We performed a retrospective cohort study including 12 patients with primary malignant sacral tumors, including 5 men and 7 women with a mean age of 58.25 years (range 20–66 years), undergoing total *en bloc* sacrectomy with 3D printed implant reconstruction from 2016 to 2021. There were 7 cases of chordoma, 3 cases of osteosarcoma, 1 case of chondrosarcoma and 1 case of undifferentiated pleomorphic sarcoma. We use CAD technology to determine surgical resection boundaries, design cutting guides, and individualized prostheses, and perform surgical simulations before surgery. The implant design was biomechanically evaluated by finite element analysis. Operative data, oncological and functional outcomes, complications, and implant osseointegration status of 12 consecutive patients were reviewed.

**Results:** The implants were implanted successfully in 12 cases without death or severe complications during the perioperative period. Resection margins were wide in 11 patients and marginal in one patient. The average blood loss was 3875 mL (range, 2000–5,000 mL). The average surgical time was 520 min (range, 380–735 min). The mean follow-up was 38.5 months. Nine patients were alive with no evidence of disease, two patients died due to pulmonary metastases, and one patient survived with disease due to local recurrence. Overall survival was 83.33% at 24 months. The Mean VAS was 1.5 (range, 0–2). The mean MSTS score was 21 (range, 17–24). Wound complications occurred in 2 cases. A deep infection occurred in one patient and the implant was removed. No implant mechanical failure was identified. Satisfactory osseointegration was found in all patients, with a mean fusion time of 5 months (range 3–6 months).

**Conclusion:** The 3D-printed custom sacral prosthesis has been effective in reconstructing spinal-pelvic stability after total *en bloc* sacrectomy with satisfactory clinical outcomes, excellent osseointegration, and excellent durability.

## 1 Introduction

Primary malignant sacral tumors are rare and include chordoma, chondrosarcoma, osteosarcoma, and Ewing sarcoma (Senne et al., 2021). En-bloc wide resection is the recommended surgical treatment for the management of sacral malignancies, which can prolong survival time (Chatain and Finn, 2020). Large bone defect after total sacrectomy resulting in spinopelvic discontinuity leads to significant instability. Unless reconstruction restores continuity and stability, the patient’s postoperative function and quality of life will be severely limited (Kim et al., 2021).

Reconstruction after a total sacrectomy is a complex procedure due to the special anatomical and biomechanical factors of the lumbosacral region. The reconstruction technique should provide sound stability, which facilitates early pain-free mobility and bone healing. Many types of spinopelvic reconstruction have been described but long-term success is limited and remains controversial (Bederman et al., 2014). Traditional methods of spinal-pelvic reconstruction do not reconstruct satisfactorily.

The use of customized 3D-printed implants for the reconstruction of severe oncologic bone defects in selected cases is increasing when the use of conventional techniques is difficult or impossible (Wang and Yang, 2021; Meng et al., 2022). Customized implants are used for spinopelvic reconstruction in complex clinical cases (Wei et al., 2017; Chatain and Finn, 2020; Peng et al., 2020). However, there are still 30% of patients with implant failure (breakage of screws and/or rods) and other defects (Wei et al., 2019), lack long-term follow-up results, and can not provide reliable clinical prognosis information for doctors, and the prosthesis needs further optimization. We previously reported that the use of a prosthesis to restore continuity after sacral GCT resection is safe and effective and facilitates better functional outcomes (Lv et al., 2020). The design concept was of an implant with porous bone-implant interfaces to connect the posterior lumbar spine, anterior spinal column, and both sides of the ilium in one step. Given the rarity of these cases, robust data on the use of prosthetic reconstruction are lacking.

Currently, to our knowledge, a 3D-printed custom-made prosthesis with a two-wing design is rare for spinopelvic reconstruction. The purpose of this study was to describe the design concept and surgical skills of the 3D-printed prosthesis in primary malignancies of the sacrum, and explore the function, complications, and osseointegration.

## 2 Materials and methods

### 2.1 Patients

We performed a retrospective cohort study including 12 patients with primary malignant sacral tumors, including 5 men and 7 women with a mean age of 58.25 years (range 20–66 years), undergoing total sacral osteotomy with 3D printed prosthesis reconstruction from 2016 to 2021.

Twelve patients presented with complaints of lumbosacral pain and eight patients had bladder and bowel symptoms. Preoperative puncture biopsies were performed to determine the pathological classification of the tumors. 7 cases were diagnosed as chordoma, 3 as osteosarcoma, 1 as chondrosarcoma, and 1 as undifferentiated pleomorphic sarcoma. This study was approved by the Medical Ethics Committee of Qilu Hospital of Shandong University. Informed consent was obtained from all participants. Details are shown in Table 1.

**TABLE 1:** Diagnoses, operative data, oncologic, functional outcomes and complications of patients.

Patient number	Age (years)	Sex	Diagnosis (stage)	Tumor level	Tumor size (cm)	Blood loss (mL)	Operative time (min)	Surgical margin	Follow-up (months)	VAS	MSTS score (%)	Metastasis	Local recurrence	Patient status	Complications	Time of osseointegration (months)
1	64	F	Chondrosarcoma	S1–S4	10	5,000	735	Marginal	62	2	18	Yes	Yes	Died of disease	wound dehiscence, recurrence	3
2	66	F	Undifferentiated pleomorphic sarcoma	S1–S3	10	3,000	720	Wide	53	1	18	No	No	No evidence of disease	None	6
3	65	M	Osteosarcoma	S1–S4	8	4,500	520	Wide	48	2	17	Yes	No	Died of disease	wound dehiscence	6
4	20	F	Osteosarcoma	S1–S3	6	2000	395	Wide	46	1	25	No	No	No evidence of disease	None	3
5	53	M	chordoma	S1–S4	7	4,000	420	Wide	45	0	23	No	No	No evidence of disease	None	6
6	56	F	chordoma	S1–S4	8	3,500	600	Wide	42	2	23	Yes	No	Alive with disease	Deep infection	6
7	60	M	chordoma	S1–S4	10	5,000	540	Wide	36	2	18	No	No	No evidence of disease	None	6
8	55	M	chordoma	S1–S4	8	3,500	480	Wide	34	1	24	No	No	No evidence of disease	None	6
9	69	F	chordoma	S1–S3	8	4,000	380	Wide	27	2	20	No	No	No evidence of disease	None	6
10	62	F	chordoma	S1–S3	10	3,500	520	Wide	21	2	23	No	No	No evidence of disease	None	6
11	64	F	Osteosarcoma	S1–S3	12	5,000	480	Wide	28	2	20	No	No	No evidence of disease	None	3
12	65	M	chordoma	S1–S3	8	3,500	450	Wide	20	1	23	No	No	No evidence of disease	None	3

### 2.2 Prosthesis design and fabrication

**FIGURE 1:**
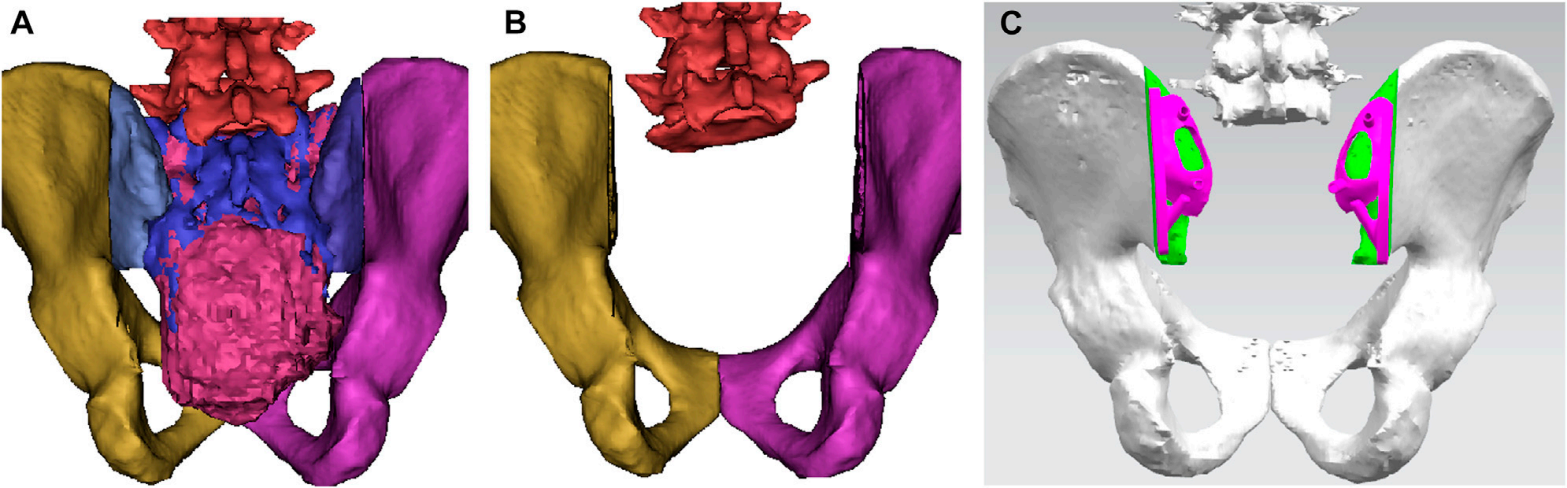
A 3D bone tumor model from CT data was created for surgical planning **(A)**. Bone defect model after tumor resection **(B)**. Design of the cutting guide **(C)**.

**FIGURE 2:**
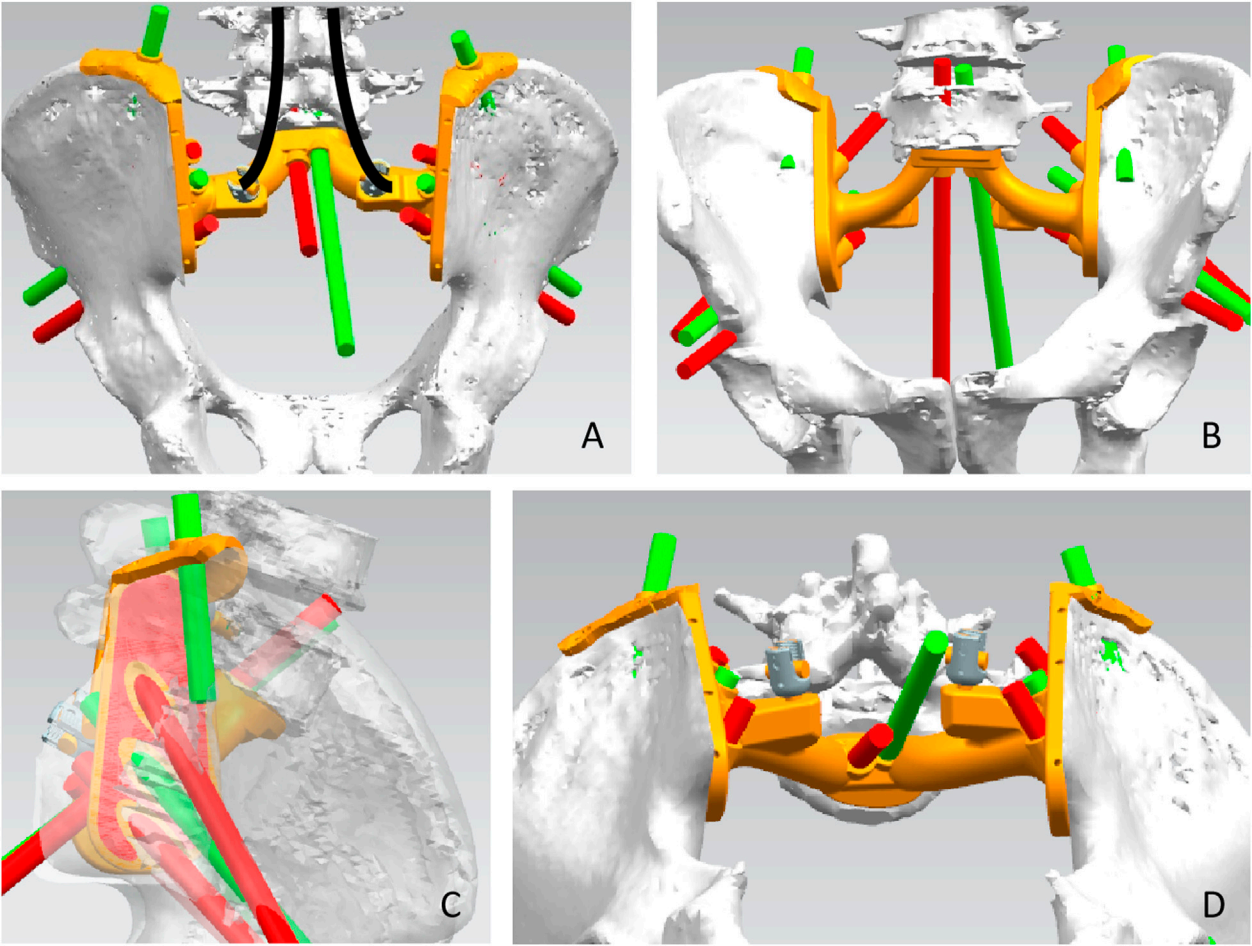
Design of the sacral implant. Dorsal view **(A)**, front view **(B)**, side view **(C)**, and upward view **(D)** of the sacral implant 3D model.

**FIGURE 3:**
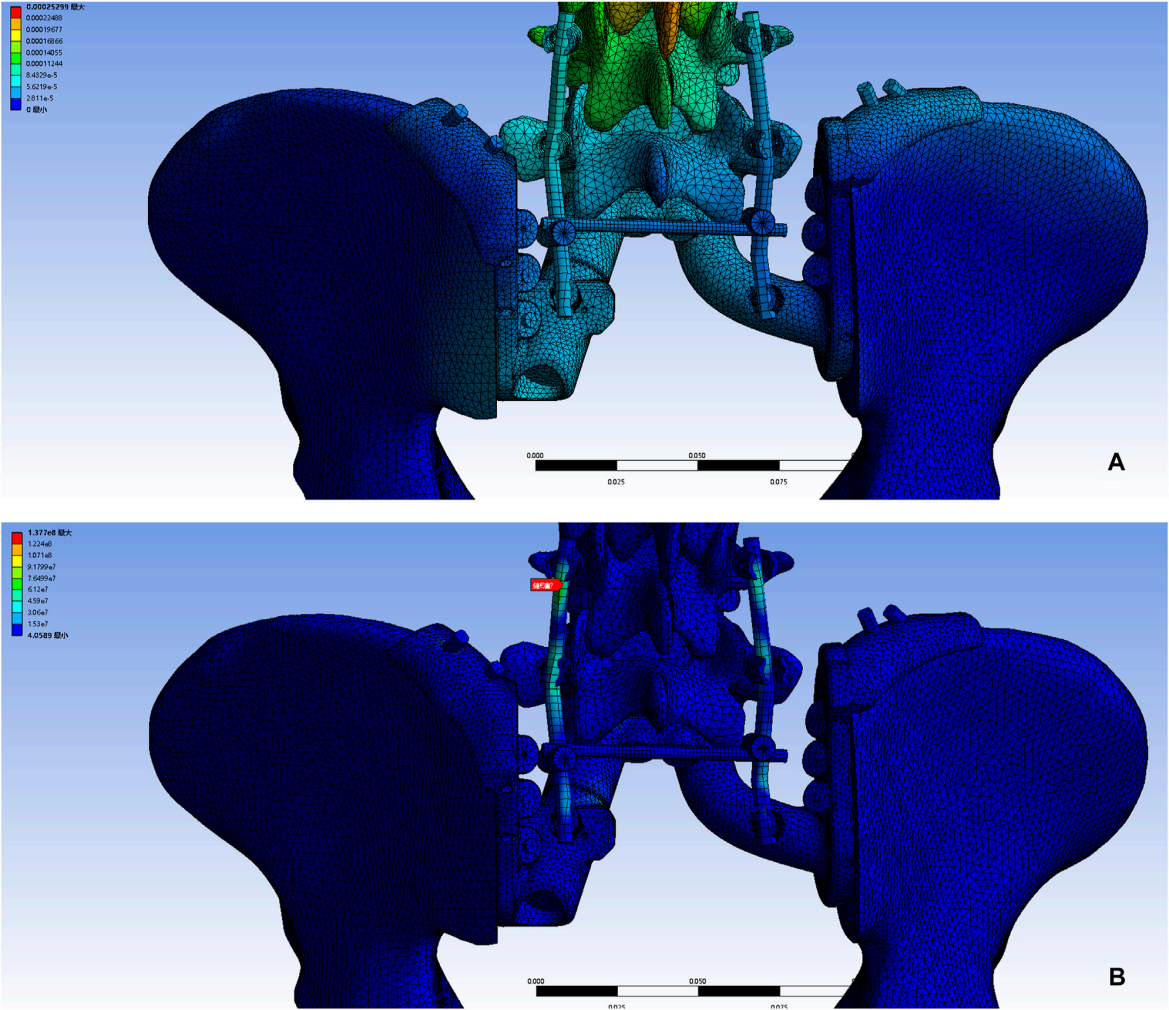
A 3D bone tumor model from CT data was created for surgical planning **(A)**. Bone defect model after tumor resection **(B)**. Design of the cutting guide **(C)**.

Pelvic CT in DICOM format was exported to the software MIMICS (Materialise, Leuven, and Belgium) to reconstruct a 3D rendering to identify anatomical details (Figure 1A). We determined the osteotomy plane and the morphology of the bone defect (Figure 1B). The osteotomy guides were highly conformed to the surface morphology of bone and had positioning holes for Kirschner wire drilling (Figure 1C). The two-wing-like sacral implant fully adapting to the bone defect was designed as a patient-specific structure (Figure 2). The central portion is attached to the lower endplate of the L5 vertebrae and the two wings are attached to the osteotomy plane of the bilateral iliac bones. The small holes facilitate the suture of the surrounding soft tissue. The lumbar pedicle screws are attached to the implant with titanium rods. The bone-implant connection is a porous structure and is firmly fixed by screws. The implant design was biomechanically evaluated by individualized finite element analysis using Abaqus (Dassault Systèmes, Velizy Villacoublay, and France) before the actual fabrication of the implant. After evaluation of the finite element analysis, we found that the bone loading neither causes fractures nor stress shielding and that the implant design is sufficiently strong (Figure 3). It takes about 2 weeks from implant design to surgery.

### 2.3 Surgical techniques

**FIGURE 4:**
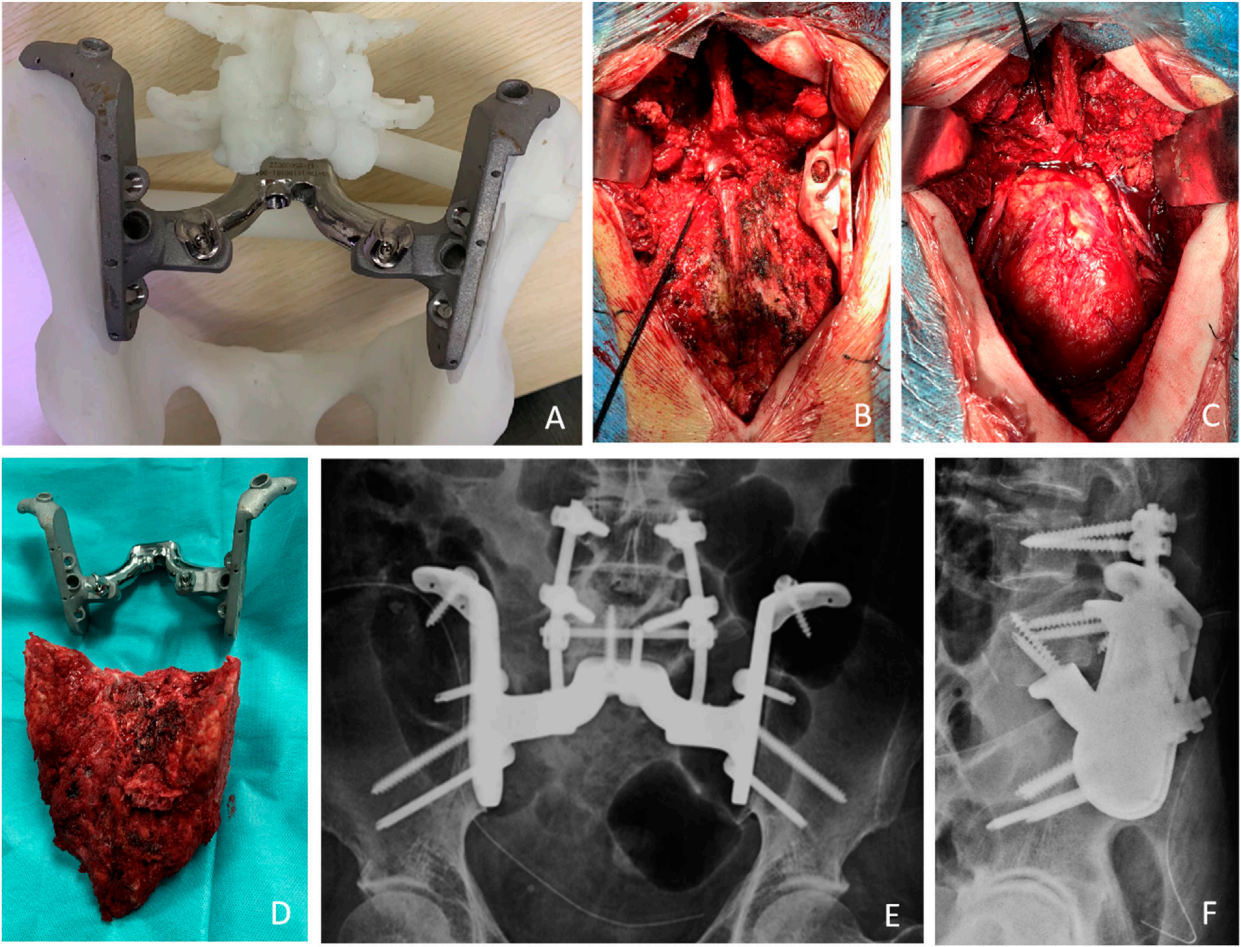
Preoperative simulation and intraoperative images. **(A)** The outer view of the implant. These models included the implant trial and the remaining bone after tumor resection allowed the surgeon to practice the procedures before the real surgery. **(B)** The cutting guide was placed according to the preoperative simulation and fixed with a Kirsch pin, the iliac bones were osteotomized bilaterally. **(C)** Bone defect after excision. **(D)** The entire sacrum was then removed in one piece along with the tumor. **(E,F)** The X-ray shows that the prosthesis has been properly placed.

Selective arterial embolization and abdominal aortic balloon occlusion were used to reduce intraoperative bleeding. An adequate preoperative enema was performed to minimize any intraoperative disturbance. An artificial vessel was prepared and if the vessel was damaged, an anastomosis was performed. After anesthesia, all patients were placed in the prone position using a posterior-only approach. The incision is an inverted Y-shaped incision. The deep fascia is incised to reach the sacrospinous muscle, exposing the dorsal sacrococcygeal, bilateral sacroiliac joints, part of the iliac crest, and the L5 spinous process. Bilateral pedicle screws are placed at L4 and L5. The sacrospinous muscle and sacral and coccygeal ligaments were removed. The space between the rectum and the sacrum is then filled with gauze and the rectum is pushed forward to ensure that the bowel wall is not damaged during the separation. The iliac vessels, ureter, sciatic nerve, and other vital structures are protected. The sacral spine was excised to expose the sacral canal and dural sac and ligated. The bilateral L5 nerve roots are carefully separated. The L5-S1 intervertebral disc is excised. A cutting guide was placed according to the preoperative simulation and fixed with a Kirsch pin, the iliac bones were osteotomized bilaterally, and the entire sacrum was then removed in one piece along with the tumor (Figure 4). A plastic implant test is used to confirm the match, followed by pulsed irrigation with isotonic sodium chloride solution, followed by a 3-min wound soak with iodophor, and then pulsed irrigation. Prosthesis installation usually begins with sublumbar endplate fixation, resetting the entire pelvis and fixing the prosthesis to the remaining iliac bone with a metal rod attached posteriorly to the lumbar spine (Figure 4). Re-irrigation is performed, followed by autograft filling with bone chips at the bone-implant interface. The soft tissue is tightly sutured to the prosthesis to reduce dead space. There was enough tissue to tightly close the wound, and we did not use a rotational or free flap in these patients.

### 2.4 Postoperative treatment and follow up

Postoperative antibiotic therapy was administered and an inflatable leg pump was used to prevent lower extremity venous thrombosis. The drainage tube was removed when the daily drainage was less than 50 mL. The length of time the catheter is left in place is determined by whether the patient can urinate. If the patient was unable to urinate, the indwelling catheter was kept in place and functional bladder exercises were continued. At 6 weeks postoperatively, patients were instructed to stand up using crutches and to perform progressive lower extremity walking exercises. One patient received adjuvant radiotherapy. Patients with osteosarcoma received adjuvant chemotherapy.

Patients have regular outpatient follow-ups for pelvic radiographic review in the third, sixth and twelfth months after surgery. After 1 year, reviews were performed every 6 months; after 3 years, reviews were performed annually. The Visual Analog Scale (VAS) was used to assess pain levels. Functional outcome was determined using the MSTS 93 system at the latest follow-up. The complications, including surgery-related complications and mechanical failures, were determined at the final follow-up. Osseointegration was assessed every 3 months using radiographs or computed tomography scans. The criterion for osseointegration is the continuous trabecular structure of the bone on the surface of the implant viewed on CT.

### 2.5 Statistical analysis

Statistical analyses were performed using IBM SPSS Statistics software, version 22 (IBM SPSS, Armonk, NY, United States). Continuous data are represented as mean.

## 3 Results

### 3.1 Operational outcomes

Resection margins were wide in 12 patients and marginal in one patient (Table 1). The average blood loss was 3,875 mL (range, 2000–5,000 mL). The average surgical time was 520 min (range, 380–735 min). No patient died of intra/perioperative complications.

### 3.2 Oncologic outcomes

At a mean follow-up of 38.5 months (range, 20–62 months), 9 patients were alive with no evidence of disease, and one patient survived with disease due to local recurrence. Two patients with osteosarcoma were found to have distant metastasis at a mean of 14.5 months postoperatively and died due to rapid tumor progression at a mean of 20.2 months. One patient had a local recurrence at 11.2 months postoperatively. Overall survival was 83.33% at 24 months.

### 3.3 Functional outcome

After surgery, all patients experienced an improvement in quality of life resulting from the reduction or resolution of pain. The Mean VAS was 1.5 (range, 0–2). All patients experienced an improvement in limb function at the final follow-up. At the last follow-up, 12 patients were able to walk independently, while 2 patients could only walk at home with walking aids. The mean MSTS score was 21 (range, 17–24). Patients all experienced postoperative bowel, bladder, and sexual function loss. After 2–6 months of bladder function exercises (mean 4 months), these patients were able to compress the bladder or pass urine on their own. Patients were instructed to perform defecation exercises, follow a controlled diet, and participate in medication-assisted therapy to defecate by regularly squeezing the lower abdomen.

### 3.4 Complications

Wound complications occurred in 2 cases as post-operative. Wound dehiscence was successfully treated with surgical wound debridement, antibiotic therapy, and VSD therapy. A deep infection occurred in one patient and the implants were removed 1 year after the operation. Local recurrence occurred in one patient.

### 3.5 Implant status

**FIGURE 5:**
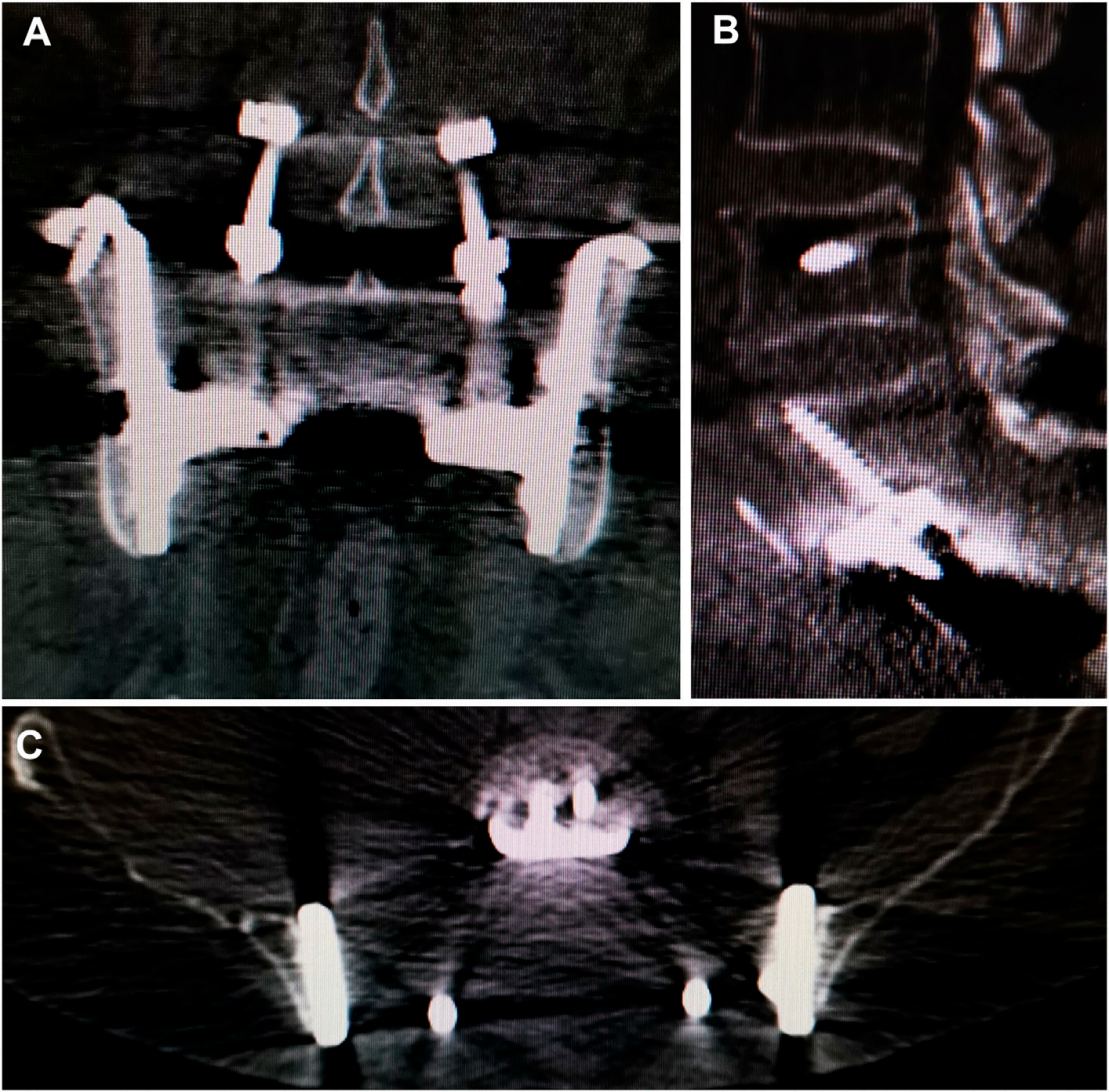
CT showed excellent osseointegration at the bone–implant junctions in coronal **(A)**, sagittal **(B)**. And axial **(C)** views.

No aseptic loosening and fracture were identied. Osseointegration at the all bone-implant interface was radiographically confirmed in all patients using CT (Figure 5).

## 4 Discussion

The treatment of extensive sacral bone loss and spinopelvic discontinuity is challenging. Spinal pelvic reconstruction after total sacrectomy is very difficult. In recent years, several studies have begun using custom-made 3D-printed prostheses in spinopelvic reconstruction, with encouraging results. Paul Wuisman et al. applied a custom-made prosthesis consisting of five components in a patient with sacral osteosarcoma involving both iliac bones, and at a 3-year postoperative follow-up, the patient was able to walk short distances outdoors with crutches (Wuisman et al., 2001). Guo Wei et al. reported a kind of total sacral reconstruction using a 3D-printed one-piece prosthesis and found that the prosthesis significantly outperformed the conventional approach in terms of reconstructive stability and motor pain function scores and that the integration between the prosthesis and bone remained strong even with the presence of broken nails (Wei et al., 2019). Doyoung Kim et al. used a 3D-printed prosthesis for reconstruction in a Hemisacrectomy, and CT at 1-year follow-up showed that bone ingrowth had occurred at the prosthesis-bone interface (Kim et al., 2017). Previously, our team used a modular prosthesis for reconstruction in the resection of sacral giant cell tumor with preservation of the sacral nerve and found that the modular design was easy to install, had excellent osseointegration properties, and helped maintain long-term stability (Lv et al., 2020). Currently, the design and application of the prosthesis are in the exploratory stage, and the surgeon’s surgical philosophy has a strong influence on the design of the prosthesis, integrated or grouped, preferring anatomical or functional reconstruction, but also showing certain commonalities, such as better matching of the bone defect structure, design of holes that can be fixed with screws, and the use of holes that facilitate lightweight and bone ingrowth. In our series, all patients regained walking function after surgery, and good bone ingrowth was found during follow-up, showing some superiority over traditional reconstruction methods. These prostheses show satisfactory results in terms of surgical technique, operating time, safety, and functional outcomes. The complication rate is comparable to other complex reconstructions.

The use of 3D printing technology in bone tumor treatment is safe and effective, reducing operative time and complication rates, obtaining satisfactory functional and oncological outcomes and has become cost-effective and reliable, making it suitable for orthopedic oncology (Yen et al., 2021). The 3D-printed prosthesis conforms to the current concept of lumbar-pelvic reconstruction (Kim et al., 2021). The prosthesis is implanted to reconstruct the anterior lumbar column and the posterior pelvic ring and is connected to the posterior lumbar spine in combination with a nail rod system to achieve all-around fixation. The 3D-printed prosthesis can be customized to fit any shape of the sacral defect. The preoperative planning and the use of osteotomy guides allow for a very good fit of the prosthesis to the bone defect. The porous structure and rough surface inside the prosthesis provide a scaffold for cellular adhesion and proliferation, and the new bone can be cross-locked inside the pores to form a strong osseointegration (Guyer et al., 2016). At our follow-up, bone osseointegration at the prosthesis-bone interface was also observed, even as the new bone shell wrapped around the edges of the prosthesis, and the L5 position and posterior pelvic ring opening remained unchanged significantly during the follow-up period, indicating very high reconstructive stability of the prosthesis. The elastic modulus of the porous structure is close to that of human cortical bone, and the elastic modulus can be adjusted by changing the structure, and porosity, or achieving a gradient porosity distribution to avoid stress fractures. In conclusion, the advantages of conforming to current reconstruction concepts, having the advantage of individualized matching, a porous structure that facilitates osseointegration, and an elastic modulus similar to that of cortical bone make 3D printed prosthesis an optimistic prospect for lumbar-pelvic stability reconstruction.

It is very important to choose the appropriate surgical approach, protect the nerves and blood vessels, avoid damaging the bowel or ureter, control the risk of bleeding, and restore stability to the lumbosacral region (Houdek et al., 2020). *En bloc* sacrectomy is a procedure with a high rate of major complications, often necessitating secondary interventions (Verlaan et al., 2015). Although most patients have permanent neurological deficits after tumor resection, extensive resection is the best way to treat sacral tumors to reduce the chance of local recurrence and prolong survival time (van Wulfften Palthe et al., 2017). Depending on the pathology of the tumor, adjuvant radiotherapy should be considered as a postoperative treatment. Unfortunately, despite total *En bloc* sacrectomy and adjuvant therapy, both older patients developed recurrence and passed away. Throughout the follow-up period, all patients steadily improved their ambulatory function and regained the ability to walk long distances to climb stairs. Patients’ MSTS scores continued to improve, reflecting the fact that the use of prosthetic reconstruction was very beneficial to the recovery of functional activity of the patient’s lower extremities. Patients in this study had a total *En bloc* sacrectomy with immediate postoperative urinary and fecal incontinence, but there was no impact on the motor ability and no loss of plantar flexion of the foot, but there was residual numbness of the lower extremity to varying degrees. A total of 2 patients experienced wound complications. Poor healing, such as wound infection and dehiscence, was reported in 29.2% of sacral tumor surgeries (Li et al., 2013). The incidence of wound infection or poor healing after resection of high sacral tumors can be 25% and 53.5% (Ruggieri et al., 2012). High suture tension at the skin margin, inadequate blood flow, and local fecal contamination are common causes. Some studies have shown that high sacral tumors, tumor volume over 200 cm^3^, and abundant tumor blood supply are independent risk factors for intraoperative hemorrhage (Tang et al., 2009). In this study, the overall bleeding was lower than that reported in the literature due to the use of preoperative embolization and balloon block to control bleeding.

Our study has some limitations. This study had a limited sample size and lacked an appropriate control group; therefore, we believe that a larger sample size, appropriate control group, and longer follow-up period are necessary. For 3D-printed prostheses, the relatively short follow-up period in this study may underestimate the potential for late complications in these patients. We consider the absence of gaps at the bone-prosthesis interface with the presence of continuous trabeculae as good osseointegration. Patients with good osseointegration did not experience displacement or screw loosening. Therefore, the impact of assessment bias was not significant. In addition, studies analyzing changes in spinal biomechanical status due to internal fixation devices could provide additional clinical evidence for optimizing treatment options. The widespread use and familiarity with the latest generation of 3D printed custom prostheses over the past 5 years or so has made this new reconstruction technique possible and therefore allows for long-term follow-up of patients. We believe the real value is the opportunity to share this experience and technical description in the hope that it will stimulate the potential for multi-institutional research and collaboration to further refine the options for this challenging clinical problem.

## 5 Conclusion

The 3D-printed custom sacral prosthesis has been effective in reconstructing spinal-pelvic stability after total *en bloc* sacrectomy with satisfactory clinical outcomes, excellent osseointegration, and excellent durability, which is worth further promotion in clinical practice.

## Data Availability

The raw data supporting the conclusion of this article will be made available by the authors, without undue reservation.
